# Reporting of patient safety incidents in minimally invasive thoracic surgery: a national registered thoracic surgeons experience for improvement of patient safety

**DOI:** 10.1093/icvts/ivac129

**Published:** 2022-05-11

**Authors:** Benjamin Bottet, Caroline Rivera, Marcel Dahan, Pierre-Emmanuel Falcoz, Sophie Jaillard, Jean-Marc Baste, Agathe Seguin-Givelet, Richard Bertrand de la Tour, Francois Bellenot, Alain Rind, Dominique Gossot, Pascal-Alexandre Thomas, Xavier Benoit D’Journo

**Affiliations:** 1 Department of General and Thoracic Surgery, Rouen University Hospital, Rouen, France; 2 Department of Thoracic Surgery, Bayonne Hospital, Bayonne, France; 3 Department of Thoracic Surgery, Larrey Hospital, CHU Toulouse, Toulouse, France; 4 Department of Thoracic Surgery, CHRU Strasbourg, Strasbourg, France; 5 Department of Thoracic surgery, Hopital Privé le Bois, Lille, France; 6 Department of Thoracic Surgery, Curie-Montsouris Thorax Institute, Institut Mutualiste Montsouris, Paris, France; 7 Paris 13 University, Sorbonne Paris Cité, Faculty of Medecine SMBH, Bobigny, France; 8 Department of Cardiovascular and Thoracic Surgery, Pontchaillou Hospital, Rennes, France; 9 Organisme d’Accréditation (OA)-CTCV, SFCTCV, Paris, France; 10 Department of Thoracic Surgery, Hopital Nord-APHM, Aix-Marseille University, Marseille, France

**Keywords:** Minimally invasive surgery, Patient safety incident, Cardiothoracic surgery, Video-assisted surgery, Lobectomy

## Abstract

**OBJECTIVES:**

The reporting of patient safety incidents (PSIs) occurring in minimally invasive thoracic surgery (MITS) is crucial. However, previous reports focused mainly on catastrophic events whereas minor events are often underreported.

**METHODS:**

All voluntary reports of MITS-related PSIs were retrospectively extracted from the French REX database for ‘in-depth analysis’. From 2008 to 2019, we retrospectively analysed and graded events according to the WHO classification of PSIs: near miss events, no harm incidents and harmful incidents. Causes and corrective measures were analysed according to the human-technology-organization triad.

**RESULTS:**

Of the 5145 cardiothoracic surgery PSIs declared, 407 were related to MITS. Among them, MITS was performed for primary lung cancer in 317 (78%) and consisted in a lobectomy in 249 (61%) patients. PSIs were: near miss events in 42 (10%) patients, no harm incidents in 81 (20%) patients and harmful incidents in 284 (70%) patients (mild: *n* = 163, 40%; moderate: *n* = 78, 19%; severe: *n* = 36, 9%; and deaths: *n* = 7, 2%). Human factors represented the most important cause of PSIs with 267/407 (65.6%) cases, including mainly vascular injuries (*n* = 90; 22%) and non-vascular injuries (*n* = 43; 11%). Pulmonary arteries were the most affected site with 57/91 cases (62%). In all, there were 7 deaths (2%), 53 patients required second surgery (13%) and 30 required additional lung resection (7%).

**CONCLUSIONS:**

The majority of reported MITS -related PSIs were non-catastrophic. Human factors were the main cause of PSIs. Systematic reporting and analysis of these PSIs will allow surgeon and his team to avoid a large proportion of them.

**Figure:**
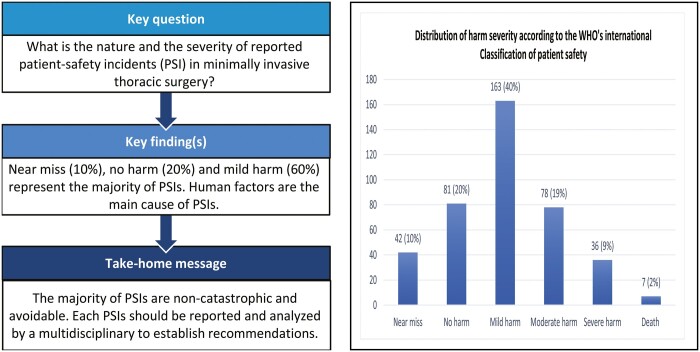

## INTRODUCTION

Advances in surgical technology have enabled the development of minimally invasive thoracic surgery (MITS). Nowadays, MITS has become the standard of care for the management of early-stage lung cancer worldwide. Video-assisted thoracic surgery (VATS) and robotic-assisted thoracic surgery (RATS) have gained increasing popularity thanks to benefits such as fewer overall complications, less pain and decreased length of hospital stay [1, 2]. However, despite these benefits, performing MITS procedures requires a high level of surgical skills to overcome the risk of technical-induced complications [3]. MITS-related catastrophic events have been reported with an incidence between 1% and 1.5% during VATS or RATS lobectomy procedures [4–6]. Fourdrain *et al.* [7, 8] showed that VATS conversion group had a higher incidence of cardiac or respiratory comorbidities compared to full-VATS group. In this setting of advanced surgical techniques, the reporting and analysis of all MITS-related patient safety incidents (PSIs) remain crucial for risk management and teaching purposes [9]. Moreover, learning from error is seen as a key indicator of quality of care [10].

However, a majority of reported adverse events with MITS have focused mainly on catastrophic situations with a clear and recognizable injury strongly affecting patient outcomes. These situations, however, represent the tip of the iceberg. The vast majority of what constitutes an MITS-related PSI is represented by trivial or minor events with a less obvious impact on patient outcomes, even though the dramatic potential is boundlessly more critical. No harm incidents (NHIs) and near miss events (NMEs) are an integral part of MITS-related PSI and are likely to represent the submerged part of the patient safety iceberg. Unfortunately, they remain underreported in routine clinical practice to avoid unnecessary litigation with patients. As a result, their exact incidence is still unknown [11–13].

The objective of this study was to investigate and describe the nature, causes, corrective measures and perioperative severity of all events contributing to MITS-related PSI.

## MATERIALS AND METHODS

### Ethics statement

The ethics committee of the French Thoracic and Cardiovascular Surgery Society (CERC-SFCTCV) approved this study (Institutional Review Board number: IRB00012919). Formal consent was not obtained due to anonymized data.

### Data availability statement

All relevant data are within the manuscript and its supporting information files.

This study was based on the French national thoracic surgery database (REX database) which collects reports of PSIs occurring during a patient’s care pathway in cardiothoracic surgery units. For the physician’s accreditation procedure, the French Health Authority has established a dedicated process by which all certified cardiothoracic surgeons (for both public and private centres) must anonymously declare at least 2 events on patient safety per year from their own practice. All events were reported according to the method developed by the Association of Litigation and Risk Management (ALARM). Between 2008 and 2019, events were prospectively declared in the REX database. All declarations were analysed by independent cardiothoracic surgeons, experts in patient safety analysis. To target some risk situations, the REX database included several risk categories. Among these risk categories, one was created in 2008 and was dedicated to ‘in-depth analysis’ of all PSI in MITS including diagnosis and treatment procedures. This category included all minimally invasive surgeries performed for benign or malignant diseases of the mediastinum, lung, oesophagus, pleura, pericardium, diaphragm and chest wall. All conventional heart and thoracic surgery, minimally invasive cardiac surgery and all events indirectly related to MITS were excluded from the present study.

According to the WHO definition [14], 3 types of MITS-related PSI are described:

- An NME is defined as ‘a patient safety incident that did not cause harm but had the potential to do so’.
- An NHI is defined as ‘a patient safety incident occurs but does not result in patient harm’.
- A harmful incident (HI) is defined as ‘a patient safety incident that resulted in harm to a patient, including harm resulting when a patient did not receive his/her planned or expected treatment’. The term ‘harmful incident’ covers what used to be known as an ‘adverse event’ and/or a ‘sentinel event’. HI was graded according to WHO classification in 4 levels (mild, moderate, severe and death). Table 1 provides the WHO classification and some examples of MITS-related PSI.

**Table 1:** Description of minimally invasive thoracic surgery-related patient safety incident according to World Health Organization classification

Incident		Definition	Examples in MITS
Near miss event		A PSI that did not cause harm but had the potential to do so	Video device breakdown but with complete replacement Damage of specimen retrieval Error in ordering material but use of other equipment Anatomical misidentification before stapling
No harm		A PSI occurs but does not result in patient harm. The outcome was not symptomatic or no symptoms were detected and no treatment was required	Bleeding of peripheral vessels requiring minimal intervention (clipping) Conversion decision before any incident Stapler locking without consequences
Harmful incident	Mild	Patient outcome was symptomatic, symptoms were mild, loss of function or harm was either minimal or intermediate but short-term and no intervention or only a minimal intervention, e.g. extra observation, resources, review or minor treatment, was required.	Conversion decision because of a minor incident Vascular injury control with conversion or not but without major bleeding Prolonged air leak not requiring reoperation Primary suture line separation fixed by a new stapling
	Moderate	Patient outcome was symptomatic, required more than a minimal intervention, e.g. additional operative procedure or additional therapeutic treatment, and/or an increased length of stay and/or caused permanent or long-term harm or loss of function.	Conversion for major intraoperative bleeding without loss of function Recurrent nerve paralysis Prolonged air leak with reoperation Reoperation for bleeding or air leak
	Severe	Patient outcome was symptomatic, required a life-saving or other major medical/surgical intervention, shortened life expectancy and/or caused major permanent or long-term harm or loss of function.	Injuries leading to additional unplanned surgery such as pulmonary artery reimplantation Phrenic nerve paralysis Stapling error leading to additional lung resection Reoperation with additional lung resection (lung necrosis)
	Death	On balance of probabilities, death was caused or brought forward in the short term by the incident.	Major intraoperative or postoperative bleeding leading to death

MITS: minimally invasive thoracic surgery; PSI: patient safety incident.

All PSIs were retrospectively analysed by 2 independent experts (XBDJ and BB) and re-classified according to the classification scheme used in road-traffic incident analysis. We used the human-technology-organization triad, represented by the surgeon (human), the material device (technology) and environment (organization) [15]. In this triad, human factors are represented by all factors related to surgeons or surgical team, technology refers to failure of instruments and surgical devices and organization includes medical environment, planning, general logistics and buildings.

### Statistical analysis

Collected data included: age, gender, body mass index, ASA classification (American society of Anesthesiologists), indication (benign, primary or metastatic tumour), type and complexity of surgery, surgeon activity, site affected by the event, causes, corrective measures taken (conversion and additional unplanned surgery) and mortality. Distal pulmonary artery is defined by segmental artery. Categorical variables were described by frequencies and percentages. Age (median, interquartile range) and body mass index (mean, standard deviation) were calculated using Microsoft^®^ Excel 2013. Graphics and tables were done using the same software.

## RESULTS

**Figure 1:**
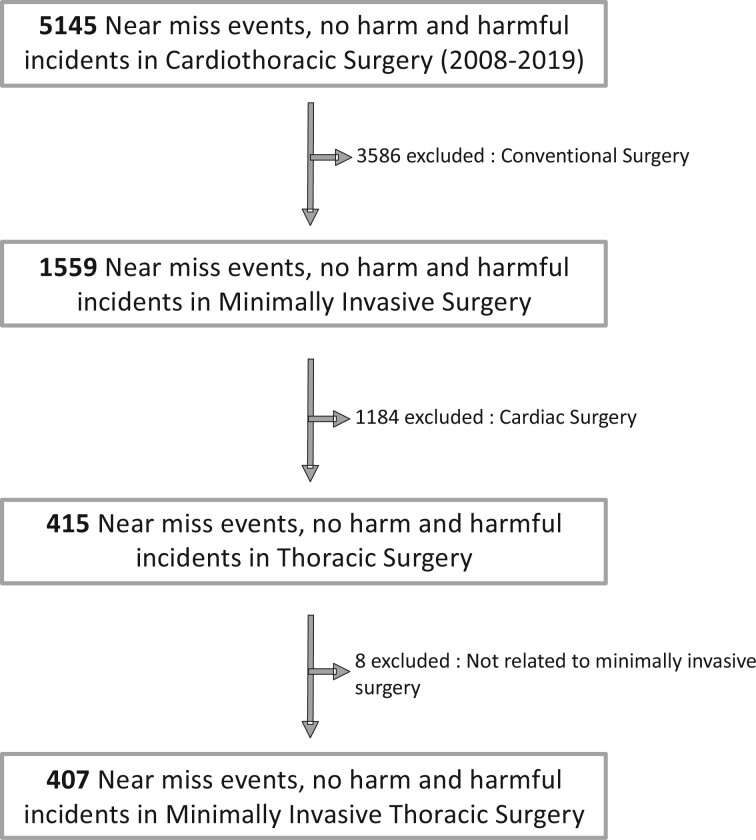
Flow chart of the study.

Of the 5145 events prospectively declared in the REX database, 1559 events were related to minimally invasive surgery and 415 to MITS. A further 8 events were excluded because they were not related to MITS. The present study is based on a total of 407 reported events (flow chart in Fig. 1).

Among the 407 patients, 259 were men (63%) and 148 were women (37%). The median age was 65 years [15–88]. Minimally invasive surgery was mainly performed for primary lung cancer in 78% (*n* = 317). Lobectomy was the most frequent procedure, *n* = 249 (61%). VATS was done for 365 patients (90%) and RATS for 42 cases (10%). The surgical procedure was declared a complex procedure by the declaring surgeon in 81 cases (20%). All patient and operative characteristics are summarized in Supplementary Material, Table S1. During the study period, 18 842 and 2021 patients were registered in the REX database as having had a VATS or an RATS procedure, respectively.

The 407 events were categorized into the human-technology-organization triad summarizing 20 clinical subcategories of NME, NHI and HI. The human category represented the most important domain with 267/407 (65%) events, followed by technology with 82/407 (20%) and organization with 58/407 (14%). Details of NME, NHI and HI are reported in Table 2. In 284/407 (70%) cases, events were considered as HI. The majority (40%) of these incidents was mild. Seven patients (2%) died. In 123/407 cases (30%), events did not compromise patient safety: 42/407 (10%) were NME and 81/407 (20%) were NHI.

**Table 2:** Classification of near miss events, no harm and harmful incidents in minimally invasive thoracic surgery

Human: surgeon	*n* = 267	%
Vascular injuries	91	22
Non-vascular injuries	43	11
Misidentification of bronchovascular structure	30	7
Pleurodesis	27	7
Position of the lung nodule	23	6
Position of the ports	22	5
Specimen retrieval	11	3
Oncology decision	8	2
Forgotten foreign body	7	2
Lobar torsion	5	1
Technology: material device	*n* = 82	%
Primary stapler malfunction	32	8
Video material device	31	8
Instruments and sterilization	19	5
Organization: environment	*n* = 58	%
Single lung ventilation	25	6
Anaesthesia	10	2
Patient installation	7	2
Team communication	6	1
Supply order	6	1
Power supply	4	1

### Vascular injuries

Vascular injuries were the most frequent events declared in the REX database (*n* = 91, 22%) leading to conversion in 79% (*n* = 72). Arteries were the most affected site with 61 cases (67%), distal and proximal pulmonary arteries represented 30 and 27 cases, respectively. Veins were 2 times less affected (*n* = 29, 32%), pulmonary veins were the most damaged (*n* = 21, 23%). Nineteen injuries (21%) were controlled without conversion. Ten cases (11%) resulted in additional unplanned surgery. Details of vascular injuries are provided in Table 3.

**Table 3:** Perioperative characteristics of vascular injury events

	*n* = 91	%
Site of injury		
Arterial	61	67
Distal pulmonary artery	30	33
Proximal pulmonary artery	27	30
Supra-aortic trunks	2	2
Bronchial artery	2	2
Venous	29	32
Pulmonary veins	21	23
Innominate vein	4	4
Superior vena cava	3	3
Subclavian vein	1	1
Heart	1	1
		
Corrective measures taken		
Conversion rate	72	79
Surgical control in VATS or RATS	19	21
Additional unplanned major surgery	10	11
Pneumonectomy	2	2
Bilobectomy	2	2
Extracorporeal membrane oxygenation	2	2
Pulmonary artery resection with end-to-end anastomosis	1	1
Heart repair	1	1
Resuscitation thoracotomy	1	1
Subclavian artery stenting	1	1

RATS: robotic-assisted thoracic surgery; VATS: video-assisted thoracic surgery.

Details of sites, causes and barriers to recovery are reported in Table 4. When injury occurred in the proximal pulmonary artery, the conversion rate was 93% (*n* = 26) compared to 72% (*n* = 21) in the distal pulmonary artery or 72% (*n* = 21) for vein injuries. Bleeding control without conversion was more frequent when the distal pulmonary artery was injured compared to the proximal pulmonary artery (52% vs 14%).

**Table 4:** Causes and corrective measures taken for the 3 most frequent sites of vascular injuries

	*n*	%
Proximal pulmonary artery		
Causes		
Dissection	12	43
Primary stapler malfunction	4	14
Direct trauma with the stapler	4	14
Coagulation	3	11
Material hanging to the staple line	2	7
Failure with surgical clips	2	7
Corrective measures taken		
Thoracotomy conversion	26	93
Bleeding control attempt in VATS or RATS	4	14
Distal pulmonary artery		
Causes		
Dissection	13	45
Coagulation	5	17
Failure with surgical clips	4	14
Direct trauma with the stapler	3	10
Material on the staple line	2	7
Primary stapler malfunction	2	7
Mishandling	1	3
Corrective measures taken		
Thoracotomy conversion	21	72
Bleeding control attempt in VATS or RATS	15	52
Pulmonary veins		
Causes		
Dissection	15	71
Primary stapler malfunction	3	14
Mishandling	2	10
Excessive traction with a vessel loop	1	5
Corrective measures taken		
Thoracotomy conversion	15	71
Bleeding control attempt in VATS or RATS	9	43

RATS: robotic-assisted thoracic surgery; VATS: video-assisted thoracic surgery.

Vascular injuries were considered as NHI in 12/91 (13%) cases and as HI in 79/91 (87%) cases (mild: 53, moderate: 11, severe: 10 and death: 5) (Supplementary Material, Fig. S1). The mortality rate was 6% (*n* = 5). Three patients died after massive intraoperative bleeding, including one patient with heart injury. Two patients died after surgery in the postoperative period, the first from an early onset of pericardial tamponade, the second from a late onset of septic shock after massive intraoperative bleeding. One patient had severe acute respiratory distress syndrome leading to a prolonged stay in intensive care unit.

### Non-vascular injuries

Non-vascular injuries were the second most frequent patient safety event reported. Non-vascular injuries were mainly diagnosed during surgery in 60% of cases but in 40% of cases, the effects of the injury were observed during the postoperative follow-up. Details of non-vascular injuries are reported in Table 5. Lymph node dissection was the main cause of non-vascular injuries (*n* = 16, 37%). Tissue dissection and use of electrocautery caused 15 events. The conversion rate was 37% (*n* = 16/43). Eight patients had to be re-operated (19%). Non-vascular injuries were considered as NHI in 5/43 (11.5%) cases and as HI in 38/43 (88.5%) cases (mild: 15, moderate: 18, severe: 5) (Supplementary Material, Fig. S2). In 5 cases, nerve injury was definitive and in 3 cases of recurrent nerve paralysis, vocal cord medialization was later performed. Two additional unplanned lung resections (1 lobectomy and 1 bilobectomy) were required.

**Table 5:** Perioperative characteristics in non-vascular injuries

	*n* = 43	%
Site of injury		
Perioperative discovery		
Tracheobronchial tree	14	33
Pulmonary parenchyma	5	12
Oesophagus	2	5
Phrenic nerve	2	5
Recurrent nerve	1	2
Coronary artery bypass	1	2
Thoracic duct	1	2
Postoperative discovery		
Pulmonary parenchyma	5	12
Phrenic nerve	4	9
Recurrent nerve	3	7
Oesophagus	1	2
Pleura	1	2
Spleen	1	2
Bronchus	1	2
Thymus	1	2
Causes		
Lymph node dissection	16	37
Mishandling	9	21
Dissection	8	19
Electrocautery of the lung	7	16
During stapling	3	7
Position of the ports	1	2
Corrective measures taken		
Thoracotomy conversion	16	37
Additional unplanned surgery	13	30
Reoperation	8	19
Medialization	3	7
Additional lung resection	2	5

### Primary stapler malfunction

Primary stapler malfunction was the main cause of incidents in the technology category. Details of site, causes and barriers are listed in Table 6. The main cause of stapler malfunction was the stapler locking (*n* = 15) which occurred mainly in parenchyma stapling (*n* = 21, 66%). Eleven patients had to be converted to repair stapler malfunction (34%). Only 2 additional surgeries were performed. One patient died in intensive care unit after major intraoperative bleeding.

**Table 6:** Perioperative characteristics in primary stapler malfunction

	*n* = 32	%
Site of injury		
Pulmonary parenchyma	21	66
Pulmonary vein	5	16
Pulmonary artery	4	13
Tracheobronchial tree	2	6
Causes		
Stapler locking	15	47
Missing staple line	7	22
Primary suture line separation	6	19
Secondary suture line separation	3	9
Wrong staple device	1	3
Corrective measures taken		
Thoracotomy conversion	11	34
Additional unplanned surgery	2	6
Reoperation	2	6

### Misidentification of bronchovascular structure

Table 7 summarizes the several misidentifications of anatomical structure. In 11 cases (37%), conversion was performed before any injury and the site of misidentification was not clearly defined.

**Table 7:** Perioperative characteristics in the misidentification of bronchovascular structure

	*n* = 30	%
Site of injury		
None	11	37
Pulmonary vein	7	23
Pulmonary artery	5	17
Bronchus	5	17
Parenchyma	1	3
Heart	1	3
Causes		
Cutting error	17	57
Anatomical variation	6	20
Difficult dissection	3	10
Unknown	2	7
Direct trauma	1	3
Failure with surgical clip	1	3
Corrective measures taken		
Thoracotomy conversion	22	73
Additional unplanned surgery	15	50
Bilobectomy	6	20
Bronchus or artery reimplantation	4	13
Lobectomy	3	10
Pneumonectomy	2	7

When the site was declared, bronchovascular structures were the most frequent: pulmonary vein (*n* = 7), pulmonary artery (*n* = 5) and bronchus (*n* = 5). More than half of the events (57%) were due to stapling error. Six variations of bronchovascular anatomy (20%) were not identified on preoperative computed tomography scan. Conversion to thoracotomy was performed in 22 cases (73%). Fifty per cent of patients had an additional unplanned surgery (*n* = 15). Additional unplanned lung resections were necessary in 11 patients (37%) due to complications: 6 completion bilobectomies, 3 completion lobectomies and 2 completion pneumonectomies. Six second surgeries were performed during the postoperative period.

### Pleurodesis

PSI was reported due to adhesions with significant injury to pulmonary parenchyma in 67% (*n* = 18). In nearly half of cases, predisposing factors were identified: previous thoracic surgery (*n* = 5), thoracic infectious disease (*n* = 4), coronary artery bypass surgery (*n* = 2) and previous mediastinal radiotherapy (*n* = 2). Twenty-one events led to conversions (78%) and 6 patients had to be re-operated. One major bleed occurred requiring a second surgery and the patient died in intensive care unit of multi-organ failure (Supplementary Material, Table S2).

### Other categories

Other risk categories are detailed in Supplementary data. An in-depth analysis was conducted and is provided in Supplementary Material.

## DISCUSSION

In this study, we report 407 voluntary declarations of several aspect of MITS-related PSI. Our data show that the vast majority of MITS-related PSI occurred after scheduled lobectomy for cancer. A small proportion of PSI was NME and NHI representing 30% compared to HI which represented 70% of all declared incidents. Human factors represent the main cause of PSI. Vascular injuries were the main causes of declarations and were associated with multiple and severe consequences. However, the findings of our study warrant further discussion.

Firstly, reports of NME and NHI may provide critical information for understanding and preventing future HI based on the fact that there is one major intraoperative event for every 19.4 NME [16]. However, identifying and analysing NME is difficult [9]. Thanks to the clear traceability of medical records HI can be retrospectively analysed. However, as NME and NHI are not harmful to patients, these cases are largely underreported. Indeed, NME and NHI are never reported in medical records in order to avoid unnecessary litigation with patients and their families. The only way to identify these PSI is to analyse anonymous and voluntary declarations in the setting of a certification procedure with the unique goal of improving patient safety. Improving the reporting of these events depends on the willingness of medical and paramedical teams to report them. According to the WHO [9], a successful reporting and learning system should have the following characteristics: reporting is safe for the individuals who report, leads to a constructive response, expertise and adequate financial resources are available to allow for meaningful analysis or reports and the reporting system must be capable of disseminating information on hazards and recommendations for changes. The important point is that a reporting system must produce a visible, useful response by the reporter to stimulate individuals or institutions to report. To date, the exact incidences of NME and of NHI remain unknown. Based on a previous study, 57% of physicians failed to report an NME whereas 7% failed to report HI [17]. The same results were found with residents [18]. Hamilton *et al.* [11] found 65 times more events with observers present in the operating room than with a specific electronic system. Our data show that nearly 30% of all PSI were NME and NHI, but the exact rate is probably much higher. The so-called ‘catastrophic events’ represented in our study by severe HIs and deaths, accounted for <10% of all reported events. A declaration bias could, however, be suspected due to surgeons’ feelings of guilt regarding those occurrences.

Secondly, our data show that human factors represent a high proportion (65%) of the global occurrence of MITS-related PSI. The term ‘human factors’ is used to describe interactions between individuals at work, the task at hand and the workplace itself. Including physical and psychological behaviour in a specific environment, some authors highlight that human factors are the main cause of PSI, representing up to 70% of events [19, 20]. The opportunity to learn from error represents a valuable source of information that can be used to teach surgical decision-making, risk management, error recovery mechanisms and team training. Failures in the operating room, particularly catastrophic ones, rarely happen as a result of a single unsafe act. Rather, they are the culmination of multiple errors involving the procedure, team, situation and organization [21]. In a report of the American Joint Commission, a lack of communication between caregivers was the main cause of nearly 70% of the thousands of adverse events reported between 1995 and 2005 [22]. Indeed, Gillespie *et al.* [23] found that inverse associations exist between the number of miscommunications and interruptions and surgical team non-technical skill score. Incivility in the operating room has a negative impact on anaesthesia trainee performance in several domains including technical skills and non-technical skills [24]. As in the aviation and the nuclear industry, the use of a standardized checklist in the operating room decreased complication and mortality rates [25]. Checklist time is a moment of sharing and checking between operating team staff before the surgical procedure [26]. Neily *et al.* [27] have shown that preoperative briefing is a key component in reducing mortality by providing a final chance to correct problems. Tschan *et al.* [28] implemented intraoperative briefings called StOP protocol allowing improvement of patient outcomes. Team communication is a central component of managing and averting errors in the operating room. That is why simulation-based training has become an integral part of surgical team training. Technical and non-technical skills learning takes place in a safe and stress-free environment. Jensen *et al.* [29] showed that virtual reality simulators for training and assessment of technical skills provided the opportunity to ensure a surgeon’s competence before performing real VATS lobectomy. Recent publications highlighted the importance of developing non-technical skills in a surgical team to improve the planning and safety of a VATS lobectomy [30] or to manage an operating room crisis [31, 32]. Team simulation and crisis resource management are innovative pedagogical tools to improve communication and behaviour. Baste *et al.* [32] developed simulation-based crisis training using models of catastrophic events in MITS. These training sessions are also an opportunity to highlight the mechanism of stress in a team and the fast spread to different team members. This emotive aspect is known to create a learning experience and enhance retention.

Thirdly, traumatic injuries represent one-third of declared events in our study, especially vascular injuries. Some authors have also reported that the intraoperative catastrophic events described in VATS and RATS were mostly vascular injuries and that the diagnosis of these events was made intraoperatively [5, 6]. In contrast, 40% of non-vascular traumatic events were diagnosed during the postoperative period within a few hours and up to several weeks after. A huge proportion of non-vascular traumatic events occurred during bronchovascular dissection or with the use of an electrocautery device, especially during lymph node dissection. Decaluwe *et al.* [5] developped several recommendations based on major catastrophic complications such as checking anatomical variation on computed tomography scan, using a ventilation test before bronchus section, …. The analysis of these PSIs showed that in most cases, these recommendations had not been applied. Our study shows that the control of vascular injuries was obtained with MITS in nearly 21%. We found that the conversion rate depends on the site of the injury when the pulmonary artery is involved. The conversion rate was 72% for the distal localization whereas it increased to 93% when the injury was proximal. The immediate management of the injury included compression and communication between anaesthesia and surgical teams. Applying pressure could be enough to control bleeding while the team is preparing itself [33]. Otherwise, a thoracotomy had to be performed when it was impossible to control bleeding, or when the injury was insufficiently exposed or when the proximal artery was involved.

Fourthly, the erroneous stapling of bronchovascular structures was an important cause of MITS-related PSI as already suggested in the literature [4–6, 13, 34]. However, this situation leads to a large and critical proportion of additional unplanned lung resections (lobectomy, bilobectomy and pneumonectomy) or bronchovascular lobectomy in nearly 50% of cases. Flores *et al.* [4] reported unplanned pneumonectomy in one of every 200 cases. We recommend always obtaining a right exposure to bronchovascular structures before stapling, and to inflate the lungs before transection. Anatomical variation on preoperative computed tomography scan should be carefully assessed [5]. Preoperative three-dimensional reconstruction [35] appears indispensable for some complex procedures and can be used as a multimodal surgical navigation system during robotic surgery in this setting [36].

### Limitations

Our study presents some important limitations that need to be acknowledged. First, the current study was not designed to provide an exhaustive description of all events that may occur in the operating room. It is only a narrative review of a declarative database of some PSI in the specific context of an individual accreditation process where the 2 main goals were an improvement of professional skills and global patient safety. Indeed, only 47% of thoracic surgeons are engaged in an accreditation process in France. The actual burden of adverse events with MITS exceeds the number of declarations. There is a 2:1 ratio between number of PSI reported and number of surgeons. However, the implementation of a declarative database is the only way to capture PSI outside a specific study done by external observers in the operating room likely resulting in much more completeness. Secondly, because of the retrospective design of our analysis, the distinction between NHI and NME was suggestive. A prospective and critical assessment by surgeons would have been much more reliable. The WHO classification of PSI must be largely diffused and all surgeons should be trained to improve PS analysis. Thirdly, this study took place over an extended period spanning the beginning of MITS to the present day in France. The improvement of surgical procedures and the development of specific equipment could have had an impact on these events.

## CONCLUSION

The majority of voluntary reported MITS-related PSI occurred after scheduled lobectomy for cancer. Very few of them (<10%) were catastrophic events and the majority were NME (10%), NHI (20%) or 60% mild to moderate HI. Human factors represent the main cause of PSI and therefore most of them could be avoidable. Among human factors, intraoperative traumatic incidents were the main cause of MITS-related PSI. Pulmonary vascular injury represents the most critical and frequent cause of incidents. Systematic declaration not only of HI but also NME and NHI should be the rule for patient safety improvement and for teaching purposes. Strong efforts in surgical and team simulation are warranted.

## SUPPLEMENTARY MATERIAL

Supplementary material is available at *ICVTS* online.

## Supplementary Material

ivac129_Supplementary_Data

## Data Availability

The data underlying this article were provided by French Health Authority (Haute Autorité de Santé—HAS) by permission. Data will be shared on request to the corresponding author with permission of French Health Authority.

## Notes

Presented at the 29th European Conference on General Thoracic Surgery - Virtual Conference, 20–22 June 2021.

## ABBREVIATIONS

HI: Harmful incident
MITS: Minimally invasive thoracic surgery
NHI: No harm incident
NME: Near miss event
PSI: Patient safety incident
RATS: Robotic-assisted thoracic surgery
VATS: Video-assisted thoracic surgery

## References

[1] Pagès P-B , Delpy J-P, Orsini B, Gossot D, Baste J-M, Thomas P et al; Epithor Project French Society of Thoracic and Cardiovascular Surgery. Propensity score analysis comparing videothoracoscopic lobectomy with thoracotomy: a French nationwide study. Ann Thorac Surg 2016 ;101:1370–8. 26872732 10.1016/j.athoracsur.2015.10.105

[2] Bendixen M , Jørgensen OD, Kronborg C, Andersen C, Licht PB. Postoperative pain and quality of life after lobectomy via video-assisted thoracoscopic surgery or anterolateral thoracotomy for early stage lung cancer: a randomised controlled trial. Lancet Oncol 2016;17:836–44. 27160473 10.1016/S1470-2045(16)00173-X

[3] Yao F , Wang J, Yao J, Hang F, Cao S, Cao Y. Video-assisted thoracic surgical lobectomy for lung cancer: description of a learning curve. J Laparoendosc Adv Surg Tech A 2017;27:696–703. 28103143 10.1089/lap.2016.0636

[4] Flores RM , Ihekweazu U, Dycoco J, Rizk NP, Rusch VW, Bains MS et al Video-assisted thoracoscopic surgery (VATS) lobectomy: catastrophic intraoperative complications. J Thorac Cardiovasc Surg 2011;142:1412–7. 22014713 10.1016/j.jtcvs.2011.09.028

[5] Decaluwe H , Petersen RH, Hansen H, Piwkowski C, Augustin F, Brunelli A et al; ESTS Minimally Invasive Thoracic Surgery Interest Group (MITIG). Major intraoperative complications during video-assisted thoracoscopic anatomical lung resections: an intention-to-treat analysis. Eur J Cardiothorac Surg 2015;48:588–98; discussion 599. 26385060 10.1093/ejcts/ezv287

[6] Cao C , Cerfolio RJ, Louie BE, Melfi F, Veronesi G, Razzak R et al Incidence, management, and outcomes of intraoperative catastrophes during robotic pulmonary resection. Ann Thorac Surg 2019;108:1498–504. 31255610 10.1016/j.athoracsur.2019.05.020 PMC6889954

[7] Fourdrain A , De Dominicis F, Iquille J, Lafitte S, Merlusca G, Witte-Pfister A et al Intraoperative conversion during video-assisted thoracoscopy does not constitute a treatment failure†. Eur J Cardiothorac Surg 2019;55:660–5. 30325413 10.1093/ejcts/ezy343

[8] Fourdrain A , Georges O, Lafitte S, Meynier J, Berna P. Intraoperative conversion during video-assisted thoracoscopy resection for lung cancer does not alter survival. Interact CardioVasc Thorac Surg 2021;33:68–75. 33585859 10.1093/icvts/ivab044 PMC8923389

[9] Larizgoitia I , Bouesseau M-C, Kelley E. WHO efforts to promote reporting of adverse events and global learning. J Public Health Res 2013;2:e29. 25170500 10.4081/jphr.2013.e29 PMC4147748

[10] Crane S , Sloane PD, Elder N, Cohen L, Laughtenschlaeger N, Walsh K et al Reporting and using near-miss events to improve patient safety in diverse primary care practices: a collaborative approach to learning from our mistakes. J Am Board Fam Med 2015;28:452–60. 26152435 10.3122/jabfm.2015.04.140050

[11] Hamilton EC , Pham DH, Minzenmayer AN, Austin MT, Lally KP, Tsao K et al Are we missing the near misses in the OR?—underreporting of safety incidents in pediatric surgery. J Surg Res 2018;221:336–42. 29229148 10.1016/j.jss.2017.08.005

[12] Fournel L , Zaimi R, Grigoroiu M, Stern J-B, Gossot D. Totally thoracoscopic major pulmonary resections: an analysis of perioperative complications. Ann Thorac Surg 2014;97:419–24. 24266953 10.1016/j.athoracsur.2013.09.091

[13] Gossot D , Lutz JA, Grigoroiu M, Brian E, Seguin-Givelet A. Unplanned procedures during thoracoscopic segmentectomies. Ann Thorac Surg 2017;104:1710–7. 28969898 10.1016/j.athoracsur.2017.05.081

[14] Cooper J , Williams H, Hibbert P, Edwards A, Butt A, Wood F et al Classification of patient-safety incidents in primary care. Bull World Health Organ 2018;96:498–505. 29962552 10.2471/BLT.17.199802 PMC6022620

[15] Warner HW , Sandin J. The intercoder agreement when using the Driving Reliability and Error Analysis Method in road traffic accident investigations. Safety Sci 2010;48:527–36.

[16] Curtis NJ , Dennison G, Brown CSB, Hewett PJ, Hanna GB, Stevenson ARL et al Clinical evaluation of intraoperative near misses in laparoscopic rectal cancer surgery. Ann Surg 2021;273:778–84. 31274657 10.1097/SLA.0000000000003452

[17] Smith KS , Harris KM, Potters L, Sharma R, Mutic S, Gay HA et al Physician attitudes and practices related to voluntary error and near-miss reporting. J Oncol Pract 2014;10:e350–7. 25095825 10.1200/JOP.2013.001353

[18] Tevis SE , Schmocker RK, Wetterneck TB. Adverse event reporting: harnessing residents to improve patient safety. J Patient Saf 2020;16:294–8. 29028690 10.1097/PTS.0000000000000333 PMC5955765

[19] Michel P , Minodier C, Lathelize M, Moty-Monnereau C, Domecq S, Chaleix P. Les événements indésirables graves associés aux soins observés dans les établissements de santé : résultats des enquêtes nationales menées en 2009 et 2004. Dossiers solidarité et santé. Dress 2010;1–18. https://drees.solidarites-sante.gouv.fr/sources-outils-et-enquetes/enquete-nationale-sur-les-evenements-indesirables-lies-aux-soins-eneis ( July 1 2020, date last accessed).

[20] Hénaux P-L , Jannin P, Riffaud L. Nontechnical skills in neurosurgery: a systematic review of the literature. World Neurosurg 2019;130:e726–36. 31284064 10.1016/j.wneu.2019.06.204

[21] Reason J. Human Error. New York: Cambridge University Press, 1990, 250 p.

[22] WHO Guidelines for Safe Surgery 2009: Safe Surgery Saves Lives [Internet]. Geneva: World Health Organization, 2009 (WHO Guidelines Approved by the Guidelines Review Committee). http://www.ncbi.nlm.nih.gov/books/NBK143243/ (10 February 2022, date last accessed).

[23] Gillespie BM , Harbeck E, Kang E, Steel C, Fairweather N, Chaboyer W. Correlates of non-technical skills in surgery: a prospective study. BMJ Open 2017;7:e014480. 10.1136/bmjopen-2016-014480 PMC5293872 28137931

[24] Katz D , Blasius K, Isaak R, Lipps J, Kushelev M, Goldberg A et al Exposure to incivility hinders clinical performance in a simulated operative crisis. BMJ Qual Saf 2019;28:750–7. 10.1136/bmjqs-2019-009598 31152113

[25] Haynes AB , Weiser TG, Berry WR, Lipsitz SR, Breizat A-HS, Dellinger EP et al A surgical safety checklist to reduce morbidity and mortality in a global population. N Engl J Med 2009;360:491–9. 19144931 10.1056/NEJMsa0810119

[26] Cooper JB. Critical role of the surgeon–anesthesiologist relationship for patient safety. Anesthesiology 2018;129:402–5. 30045093 10.1097/ALN.0000000000002324

[27] Neily J , Mills PD, Young-Xu Y, Carney BT, West P, Berger DH et al Association between implementation of a medical team training program and surgical mortality. JAMA 2010;304:1693–700. 20959579 10.1001/jama.2010.1506

[28] Tschan F , Keller S, Semmer NK, Timm-Holzer E, Zimmermann J, Huber SA et al Effects of structured intraoperative briefings on patient outcomes: multicentre before-and-after study. Br J Surg 2021;109:136–44. 34850862 10.1093/bjs/znab384 PMC10401893

[29] Jensen K , Bjerrum F, Hansen HJ, Petersen RH, Pedersen JH, Konge L. Using virtual reality simulation to assess competence in video-assisted thoracoscopic surgery (VATS) lobectomy. Surg Endosc 2017;31:2520–8. 27655381 10.1007/s00464-016-5254-6

[30] Gjeraa K , Mundt AS, Spanager L, Hansen HJ, Konge L, Petersen RH et al Important non-technical skills in video-assisted thoracoscopic surgery lobectomy: team perspectives. Ann Thorac Surg 2017;104:329–35. 28587738 10.1016/j.athoracsur.2017.03.010

[31] Bierer J , Memu E, Leeper WR, Fortin D, Fréchette E, Inculet R et al Development of an in situ thoracic surgery crisis simulation focused on nontechnical skill training. Ann Thorac Surg 2018;106:287–92. 29499178 10.1016/j.athoracsur.2018.01.058

[32] Baste J-M , Bottet B, Selim J, Sarsam M, Lefevre-Scelles A, Dusseaux M-M et al Implementation of simulation-based crisis training in robotic thoracic surgery: how to improve safety and performance? J Thorac Dis 2021;13:S26–34. 34447589 10.21037/jtd-2020-epts-03 PMC8371544

[33] Safdie FM , Sanchez MV, Sarkaria IS. Prevention and management of intraoperative crisis in VATS and open chest surgery: how to avoid emergency conversion. J Vis Surg 2017;3:87. 29078649 10.21037/jovs.2017.05.02 PMC5638443

[34] Gossot D , Merlusca G, Tudor A, Boudaya MS, Radu C, Magdeleinat P. Pitfalls related to the use of endostaplers during video-assisted thoracic surgery. Surg Endosc 2009;23:189–92. 18322748 10.1007/s00464-008-9765-7

[35] Seguin-Givelet A , Grigoroiu M, Brian E, Gossot D. Planning and marking for thoracoscopic anatomical segmentectomies. J Thorac Dis 2018;10:S1187–94. 29785293 10.21037/jtd.2018.02.21 PMC5949389

[36] Baste JM , Soldea V, Lachkar S, Rinieri P, Sarsam M, Bottet B et al Development of a precision multimodal surgical navigation system for lung robotic segmentectomy. J Thorac Dis 2018;10:S1195–204. 29785294 10.21037/jtd.2018.01.32 PMC5949399

