# Supplementary material for: Reporting of patient safety incidents in minimally invasive thoracic surgery: a national registered thoracic surgeons experience for improvement of patient safety
Source: Interact Cardiovasc Thorac Surg. 2022 May 11;35(3):ivac129. doi: 10.1093/icvts/ivac129 (PMC9419675; doi:10.1093/icvts/ivac129)

Figure S1 : Distribution of harm severity according to the WHO's internationnal Classification of patient safety for vascular injuries

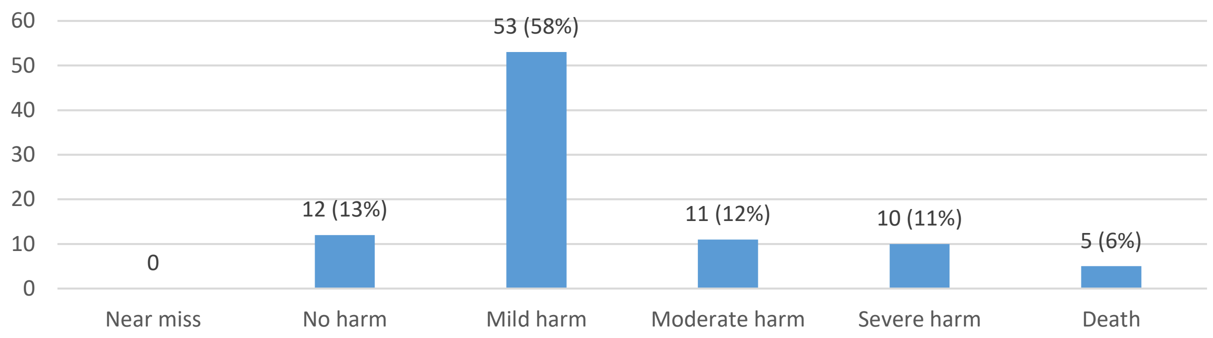

Figure S2 : Distribution of harm severity according to the WHO's internationnal Classification of patient safety for non vascular injuries

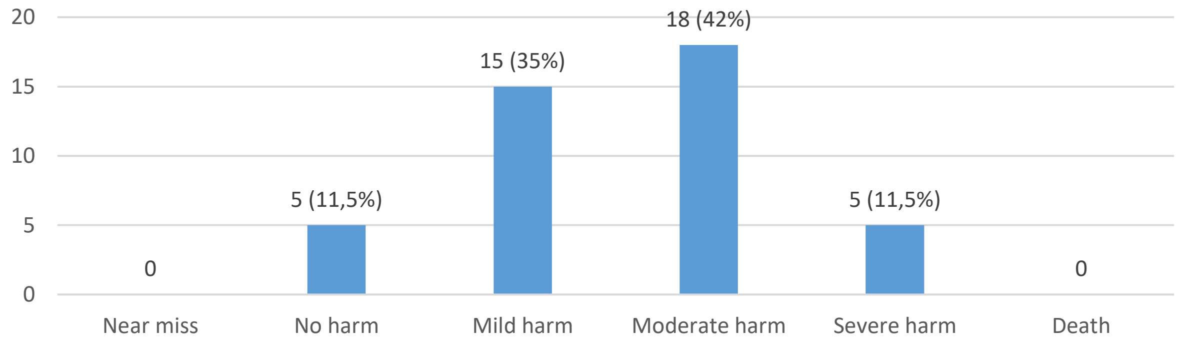

Figure S3 : Distribution of harm severity according to the WHO's international Classification of patient safety for RATS procedure

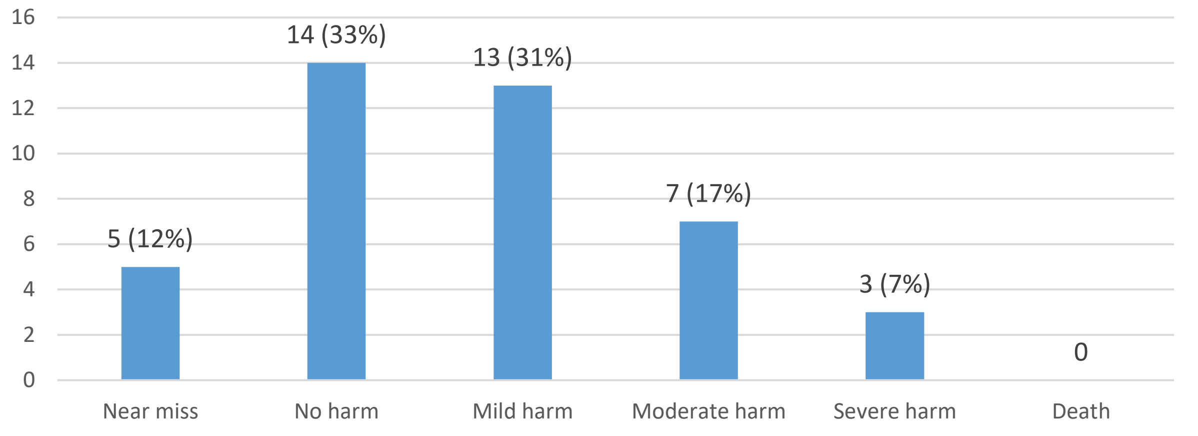

Figure S4: Distribution of harm severity according to the WHO's international Classification of patient safety for VATS procedure

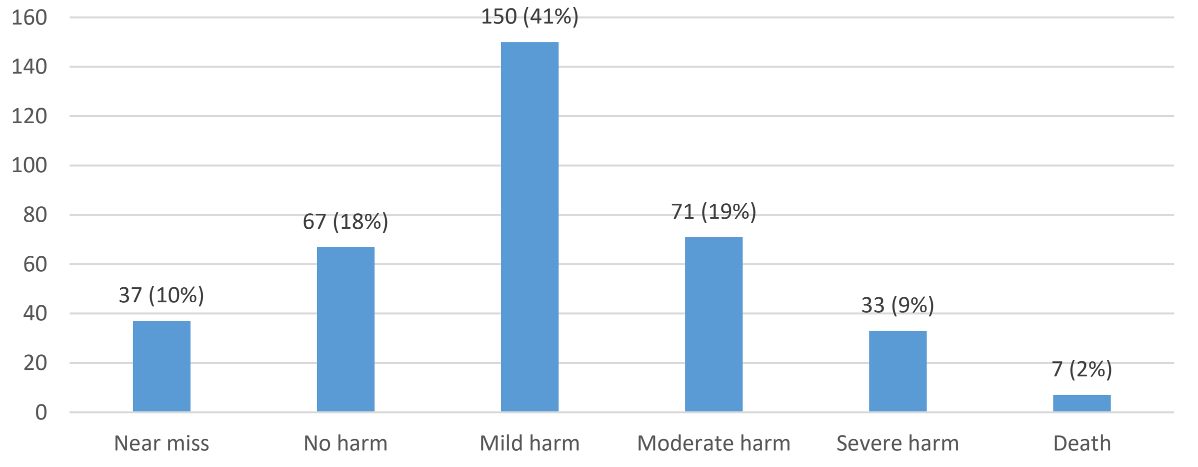

Supplement: ivac129_Supplementary_Data [file ivac129_supplementary_data.zip › ivac129_Supplementary_Data/Figures S1-S2-S3-S4.pdf]
