# Supplementary material for: Reporting of patient safety incidents in minimally invasive thoracic surgery: a national registered thoracic surgeons experience for improvement of patient safety
Source: Interact Cardiovasc Thorac Surg. 2022 May 11;35(3):ivac129. doi: 10.1093/icvts/ivac129 (PMC9419675; doi:10.1093/icvts/ivac129)
Supplement: ivac129_Supplementary_Data [file ivac129_supplementary_data.zip › ivac129_Supplementary_Data/Supplementary Data.docx]

**Supplementary Data**

| **Table S1: Patient and operative characteristics** | | |
| --- | --- | --- |
|  | n | % |
| **Age (years), median (IQR)** | 65 (56-71) |  |
| **Gender** |  |  |
| Male | 259 | 63 |
| Female | 143 | 37 |
| **BMI, mean (SD)** | 26.7 (5.5) |  |
| **ASA classification** |  |  |
| I | 160 | 39 |
| II | 153 | 38 |
| III | 91 | 22 |
| IV-V | 3 | 1 |
| **Operating indication** |  |  |
| Primary lung cancer | 317 | 78 |
| Benign pleural pathology | 36 | 9 |
| Metastatic lung cancer | 24 | 6 |
| Mediastinal pathology | 24 | 6 |
| Others | 6 | 1 |
| **Operating procedure** |  |  |
| Lobectomy | 249 | 61 |
| Wedge | 62 | 15 |
| Pleural surgery | 35 | 9 |
| Mediastinal Surgery | 24 | 6 |
| Segmentectomy | 23 | 6 |
| Mediastinoscopy | 8 | 2 |
| Others | 6 | 1 |
| RATS procedure | 42 | 10 |
| **Complexity of the procedure** |  |  |
| Very low | 185 | 45 |
| Low | 141 | 35 |
| High | 79 | 19 |
| Very high | 2 | 1 |
| **Surgeon activity** |  |  |
| Private | 115 | 41 |
| Public | 99 | 36 |
| Both (public and private) | 61 | 22 |
| Military | 2 | 1 |

| **Table S2: Perioperating characteristics in pleurodesis** | | |
| --- | --- | --- |
|  | n=27 | % |
| **Site of injury** |  |  |
| Parenchyma | 18 | 67 |
| Pleura | 4 | 15 |
| None | 3 | 11 |
| Internal mammary artery | 1 | 4 |
| Muscle | 1 | 4 |
|  |  |  |
| **Contributing factors** |  |  |
| None | 11 | 41 |
| Previous thoracic surgery | 5 | 19 |
| Infectious disease | 4 | 15 |
| Low experience in VATS or RATS | 3 | 11 |
| Coronary artery bypass | 2 | 7 |
| Previous mediastinal radiotherapy | 2 | 7 |
|  |  |  |
| **Corrective measures** |  |  |
| Thoracotomy conversion | 21 | 78 |
| Additional unplanned surgery | 6 | 22 |
| Re-operation | 6 | 22 |

| **Table S3: Characteristcs of events related to video material device** | | |
| --- | --- | --- |
|  | n=31 | % |
| **Device breakdown** |  |  |
| Camera | 12 | 39 |
| Video components (hard drive, screen, motherboard, etc.) | 9 | 29 |
| Fiber optic light cable | 5 | 16 |
| Optical sheath | 3 | 10 |
| Unavailability of video material device | 1 | 3 |
| Robotic equipment | 1 | 3 |
|  |  |  |
| **Corrective measures taken** |  |  |
| Conversion | 5 | 16 |

| **Table S4: Characteristics of events related to single lung ventilation** | | |
| --- | --- | --- |
|  | n=25 | % |
| **Reported events** |  |  |
| No single lung ventilation | 21 | 84 |
| Endotracheal intubation failure | 1 | 4 |
| Wrong endotracheal tube | 1 | 4 |
| Failure with pulmonary re-expansion | 1 | 4 |
| Logout ventilator | 1 | 4 |
|  |  |  |
| **Contributing factors** |  |  |
| No available bronchoscope | 6 | 24 |
| Inexperienced anesthesiologists | 3 | 12 |
| Difficult endotracheal intubation | 1 | 4 |
| Nurse anesthesia student alone | 1 | 4 |
| Poor fixation of endotracheal blocker | 1 | 4 |
| No training with bronchoscopy | 1 | 4 |
|  |  |  |
| **Corrective measures taken** |  |  |
| Conversion | 15 | 60 |
| Re-operation | 4 | 16 |

| **Table S5: Characteristics of events related to instrumentation and sterilization** | | |
| --- | --- | --- |
|  | n=25 | % |
| **Reported events** |  |  |
| Broken instrument | 7 | 28 |
| Sterilization fault | 5 | 20 |
| Error in ordering material | 5 | 20 |
| Optic light cable sheath defect | 3 | 12 |
| Lack of material | 3 | 12 |
| Lack of CO2 insufflator | 1 | 4 |
| Material defect | 1 | 4 |
|  |  |  |
| **Corrective measures taken** |  |  |
| Conversion | 5 | 20 |

| **Table S6: Characteristics of events related to position of lung nodule** | | |
| --- | --- | --- |
|  | n=23 | % |
| **Reported events** |  |  |
| Lung nodule not found | 10 | 43 |
| Erroneous lung resection | 5 | 22 |
| Tracking failure | 4 | 17 |
| No single lung ventilation | 2 | 9 |
| Incomplete nodule resection | 2 | 9 |
|  |  |  |
| **Corrective measures taken** |  |  |
| Conversion | 7 | 30 |
| Additional unplanned surgery | 9 | 39 |
| Additional lung resection | 7 | 30 |
| Re-operation | 1 | 4 |
| Post-operative radio-frequency ablation | 1 | 4 |

| **Table S7: Characteristics of events related to port position** | | |
| --- | --- | --- |
|  | n=22 | % |
| **Site of injury** |  |  |
| **Per-operative discovery** |  |  |
| Diaphragm | 4 | 18 |
| Liver | 3 | 14 |
| Pulmonary artery | 1 | 5 |
| Rib | 1 | 5 |
| Lung parenchyma | 1 | 5 |
| Intercostal vein | 1 | 5 |
| Pleura | 1 | 5 |
| Not applicable | 3 | 14 |
| **Post-operative discovery** |  |  |
| Muscle | 7 | 32 |
|  |  |  |
| **Causes** |  |  |
| Wrong position | 11 | 50 |
| Wound closure | 5 | 23 |
| Material misuse | 3 | 14 |
| Bleeding | 2 | 9 |
| No single lung ventilation | 1 | 5 |
|  |  |  |
| **Corrective measures taken** |  |  |
| Conversion | 7 | 32 |
| Additional unplanned surgery | 6 | 27 |
| Parietal hernia | 4 | 18 |
| Haemothorax | 2 | 9 |

| **Table S8: Characteristics of events related to specimen retrieval** | | |
| --- | --- | --- |
|  | n=11 | % |
| **Reported events** |  |  |
| Endobag rupture | 8 | 73 |
| Specimen rupture | 2 | 18 |
| Wrong size of endobag | 1 | 9 |
|  |  |  |
| **Contributing factors** |  |  |
| New material device | 2 | 18 |
| Trainee surgeon | 1 | 9 |
| No single lung ventilation | 1 | 9 |
| Bulky tumour | 1 | 9 |
|  |  |  |
| **Corrective measures taken** |  |  |
| Conversion | 2 | 18 |
| Additional unplanned surgery | 1 | 9 |
| Chest wall resection | 1 | 9 |

| **Table S9: Characteristics of events related to anesthesia** | | |
| --- | --- | --- |
|  | n=10 | % |
| **Reported events** |  |  |
| Intra-luminal foreign body | 5 | 50 |
| Difficulty with tracheal tube | 3 | 30 |
| Bilateral pneumothorax | 1 | 10 |
| Iterative tracheal tube changes | 1 | 10 |
|  |  |  |
| **Contributing factors** |  |  |
| Communication problem | 3 | 30 |
| Lack of material | 1 | 10 |
|  |  |  |
| **Corrective measures taken** |  |  |
| Conversion | 6 | 60 |
| Additional unplanned surgery | 8 | 80 |
| Bronchus resection with termino-terminal anastomosis | 4 | 40 |
| Lobectomy | 2 | 20 |
| Esophagus repair | 1 | 10 |
| Diaphragmatic repair | 1 | 10 |

| **Table S10: Characteristics of events related to oncological decision** | | |
| --- | --- | --- |
|  | n=8 | % |
| **Site of injury** |  |  |
| Bronchus | 2 | 25 |
| Parenchyma | 2 | 25 |
| Pleura | 1 | 12.5 |
| Pulmonary artery | 1 | 12.5 |
| Not applicable | 2 | 25 |
|  |  |  |
| **Causes** |  |  |
| Incomplete resection | 3 | 38 |
| Increase in tumour size | 2 | 25 |
| Lymph node extension | 1 | 12.5 |
| Oncological doubt | 1 | 12.5 |
|  |  |  |
| **Corrective measures taken** |  |  |
| Conversion | 4 | 50 |
| Additional unplanned surgery | 3 | 38 |
| Bilobectomy | 1 | 13 |
| Wedge | 1 | 13 |
| Re-operation | 1 | 13 |

| **Table S11: Characteristics of events related to forgotten foreign body** | | |
| --- | --- | --- |
|  | n=7 | % |
| **Corrective measures taken** |  |  |
| Conversion | 2 | 29 |
| Re-operation | 2 | 29 |

| **Table S12: Characteristics of events related to patient installation** | | |
| --- | --- | --- |
|  | n=7 | % |
| **Reported events** |  |  |
| Improper patient installation | 3 | 43 |
| Unexpected movement of the operating table | 2 | 29 |
| Improper surgeon installation | 1 | 14 |
| Improper robotic console installation | 1 | 14 |
|  |  |  |
| **Consequences** |  |  |
| Nerve injury | 2 | 29 |
| Sacrococcygeal dislocation | 1 | 14 |

| **Table S13: Characteristics of events related to team communication** | | |
| --- | --- | --- |
|  | n=6 | % |
| **Reported events** |  |  |
| Wrong stapling line | 2 | 33 |
| Forgetting lymph node dissection | 2 | 33 |
| Lack of material | 1 | 17 |
| Unintentional loss of CO2 | 1 | 17 |
|  |  |  |
| **Corrective measures taken** |  |  |
| Conversion | 0 | 0 |
| Additional unplanned surgery | 1 | 17 |
| Lymph node dissection | 1 | 17 |

| **Table S14: Characteristics of events related to supply order** | | |
| --- | --- | --- |
|  | n=6 | % |
| **Causes** |  |  |
| Depletion of stapling line stock | 6 | 100 |
|  |  |  |
| **Corrective measures taken** |  |  |
| Conversion | 1 | 17 |

| **Table S15: Characteristics of events related to lobar torsion** | | |
| --- | --- | --- |
|  | n=5 | % |
| **Site of torsion** |  |  |
| Middle lobe | 2 | 40 |
| Left lower lobe | 2 | 40 |
| Left upper lobe | 1 | 20 |
|  |  |  |
| **Corrective measures taken** |  |  |
| Conversion | 4 | 80 |
| Additional unplanned surgery | 3 | 60 |
| Pneumonectomy | 2 | 40 |
| Re-operation | 1 | 20 |

| **Table S16: Characteristics of events related to power supply** | | |
| --- | --- | --- |
|  | n=4 | % |
| **Reported events** |  |  |
| Brief blackout | 2 | 50 |
| Video material device power outage | 2 | 50 |
|  |  |  |
| **Corrective measures taken** |  |  |
| Conversion | 0 | 0 |

| **Table S17: Distribution of the complexity of the procedure according to the surgical procedure** | | | | | | | | |
| --- | --- | --- | --- | --- | --- | --- | --- | --- |
| **Surgical procedure Complexity of the procedure** | Lobectomy | Wedge | Pleural surgery | Mediastinal  surgery | Segmentectomy | Mediastinoscopy | Others | **Total (n)** |
| Very low | 107 | 36 | 16 | 11 | 11 | 2 | 2 | **185** |
| Low | 94 | 16 | 9 | 10 | 9 | 3 | 0 | **141** |
| High | 48 | 10 | 8 | 3 | 3 | 3 | 4 | **79** |
| Very high | 0 | 0 | 2 | 0 | 0 | 0 | 0 | **2** |
| **Total (n)** | **249** | **62** | **35** | **24** | **23** | **8** | **6** |  |
